# A goat-derived gamma-tubulin antibody for triple-channel imaging of primary cilia

**DOI:** 10.1186/s12860-026-00595-7

**Published:** 2026-05-28

**Authors:** Eliza Karapetian, Christoph Gerhardt, Natalia Lemanska, Monia Budnik, Erik Stahl, Jonas Füner, Stefan Hirschberg, Thorsten Pfirrmann

**Affiliations:** 1 Health and Medical University, Potsdam, Germany; 2 Preclinics Gesellschaft für präklinische Forschung mbH, Potsdam, Germany; 3 Preclinics Certified products GmbH, Potsdam, Germany; 4 Behring Campus Eystrup GmbH, Eystrup, Germany; 5 Clinic for Heart Surgery (UMH), Martin-Luther-University Halle-Wittenberg, Halle (Saale), Germany

**Keywords:** Primary cilium, Ciliopathies, Immunofluorescence, Multiplex imaging, Antibody generation, Gamma tubulin, Centrosome, dSTORM microscopy

## Abstract

**Background:**

Primary cilia are essential sensory organelles, and their dysfunction leads to a broad spectrum of diseases known as ciliopathies. Immunofluorescence imaging of ciliary substructures is a key tool in cilia research, but multiplex staining is often limited by the availability of antibodies from different host species.

**Methods:**

We developed a novel goat-derived gamma tubulin antibody for basal body labelling and validated its specificity through triple-label immunofluorescence in different cell lines.

**Results:**

The antibody showed strong, specific staining of basal bodies under methanol fixation, enabling clear visualization in combination with rabbit- and mouse-derived antibodies.

**Conclusions:**

This antibody expands multiplex imaging capabilities for ciliary substructures, supporting both cilia research and diagnostics of ciliopathies.

**Supplementary Information:**

The online version contains supplementary material available at 10.1186/s12860-026-00595-7.

## Background

Primary cilia are essential cellular organelles present on the surface of various vertebrate cell types, playing key roles in numerous signaling pathways and developmental processes [1–4]. Primary cilia protrude from the cell surface and consist of several distinct components: the ciliary membrane, basal body (BB), transition zone (TZ), and axoneme. The basal body, originating from the mother centriole, functions as the organizing centre and anchoring site crucial for cilium formation [5]. Distally, adjacent to the basal body is the TZ, which operates as a diffusion barrier, precisely regulating protein trafficking into and out of the cilium [6–8]. Extending from the basal body and surrounded by the ciliary membrane is the axoneme, typically composed of nine pairs of microtubules arranged in a circular array. These microtubules provide structural support and act as tracks for molecular motors and proteins essential for cilium assembly, maintenance, and functional integrity [9, 10]. Embedded within the ciliary membrane are diverse receptor proteins responsible for detecting various stimuli, such as nutrients, morphogens, and mechanical signals, thereby influencing critical cellular processes including proliferation, differentiation, and polarity [2, 3, 11, 12].

Dysfunctional primary cilia underlie a diverse group of hereditary disorders known as ciliopathies, which exhibit organ-specific manifestations or syndromic phenotypes [4, 12, 13]. Classic ciliopathies display significant clinical heterogeneity, including conditions ranging from kidney disease and retinal degeneration to skeletal anomalies, brain malformations, and severe obesity [12, 14]. The genetic causes of ciliopathies are similarly diverse, with mutations identified in approximately 200 genes so far [15]. Structural abnormalities, including shortened or absent cilia, are commonly observed in ciliopathies and include reduced ciliary length in Joubert syndrome [13]. Mislocalization of ciliary proteins, such as ARL13B or IFT88, is often indicative of transport defects, as seen in Bardet-Biedl syndrome [16]. Additionally, abnormalities in basal body positioning or clustering can disrupt ciliogenesis, like in Meckel-Gruber syndrome [6]. Mislocalization of signaling components like Smoothened, Patched or Gli proteins in Hedgehog signaling can also cause ciliopathies [3, 17]. Emerging evidence also implicates ciliary dysfunction in additional pathologies such as cancer [18–21], metabolic disorders including obesity [22], diabetes [23–25], liver fibrosis [26], and even organismal ageing [27].

Consequently, it is important to determine and classify these various underlying pathomechanisms, and immunofluorescence techniques serve as powerful diagnostic tools to complement genetic testing [28]. The presence, absence, and length of cilia, along with the abundance of ciliary proteins, are common biomarkers for diagnosing ciliopathies [29], where specific ciliary proteins are labelled to visualise ciliary subcompartments, to study colocalization or to measure protein amount in subcompartments using densitometry [7]. Gamma-tubulin is a critical component of centrioles and basal bodies and consequently plays an essential role in ciliary assembly and disassembly as well as in microtubule organization at the microtubule-organizing centre (MTOC) [30–32]. Specific gamma-tubulin antibodies are commonly used to study such processes and to label basal bodies.

In 2016, Santa Cruz Biotechnology discontinued production and sales of goat derived polyclonal antibodies, including gamma tubulin (sc-7396), and alternative products that adequately compensate for this loss are currently unavailable. To address this gap, we generated a goat-derived gamma tubulin antibody optimized for immunofluorescence imaging of basal bodies and centrioles. This antibody enables multiplex staining in combination with commercially available antibodies raised in other host species and consequently increases the flexibility in antibody variability.

## Methods

### Sample preparation and imaging

NIH-3T3 cells (ATCC Cat# CRL-6442, RRID: CVCL_0594) or mouse embryonic fibroblasts [MEFs; obtained from murine embryos at embryonic day (E)12.5] were maintained in Dulbecco’s modified Eagle’s medium (DMEM) supplemented with 10% (v/v) fetal calf serum (FCS) and 4500 mg/l glucose (high concentration) if not mentioned otherwise. For detection of primary cilia, cells were grown to confluency and serum-starved with medium containing 0.5% FCS for at least 24 h on glass coverslips (Epredia, P231.1). Cells were fixed either with 100% methanol (MeOH) for 5 min at -20 °C or with 4% paraformaldehyde (PFA) for 60 min at 4 °C for labelling with the novel gamma-tubulin antibody. Subsequently, rinsed three times with PBS to remove methanol or PFA remnants, followed by permeabilization with PBS/0.5% Triton X‐100 for 10 min (no shaking). After three washes with PBS/0.1% Triton X‐100, cells were blocked at room temperature in PBS/0.1% Triton X‐100 containing 10% donkey serum for 1 h. Diluted primary antibodies in PBS/0.1% Triton X‐100, 1% blocking solution were incubated overnight at 4 °C. After three washing steps with PBS/0.1% Triton X‐100, incubation with secondary antibody in PBS/0.1% Triton X‐100 blocking solution was performed 3 times at room temperature for 10 min and subsequent mounting with Mowiol (ROTH, #0713.2). For immunofluorescence on mouse limb buds, mouse embryos were fixed at embryonic day (E)12.5 in 4% PFA for 1.5 h and incubated in 30% sucrose (in PBS) overnight at 4 °C. Afterwards, these were embedded in Tissue‐Tek O.C.T. compound (Sakura Finetechnical #4583) and stored at -80 °C. Transverse cryostat Sect.  (7 μm in thickness) were prepared, washed with PBS, postfixed in 100% MeOH at -20 °C for 5 min and permeabilised with PBS/0.5% Triton X‐100 for 10 min. Blocking was performed with 10% donkey serum in PBST (blocking solution) for 1 h. Afterwards, the sections were incubated in the primary antibodies [rabbit anti-Arl13b (Proteintech #17711-1-AP) and goat anti-γ-tubulin (preclinics)] diluted in blocking solution (dilution: 1:100 each) overnight at 4 °C. After three washing steps, sections were incubated with the secondary antibodies [donkey anti-rabbit Cy3 (Jackson ImmunoResearch #711-165-152) and donkey anti-goat Dylight405 (Jackson ImmunoResearch #705-475-003)] diluted in blocking solution; dilution: 1:100 each for 2 h, washed again and embedded in Mowiol.

For control experiments with blocking peptide, anti–γ-tubulin antibody (Preclinics Certified Products, D-0007-100UG; dilution 1:500) was pre-incubated overnight at 4 °C with antibody blocking peptide (1 mg/mL, Biorbyt, TUBQ1, orb218060_2). Methanol-fixed NIH3T3 cells were blocked with 10% donkey serum for 1 h at room temperature prior to incubation. Arl13b antibody (Proteintech, 17711-1-AP; dilution 1:100) and 1% donkey serum were then added to the antibody master mix. Coverslips containing cells were incubated overnight at 4 °C in a 24-well plate with the primary antibody solution containing the blocking peptide. Afterwards, cells were washed three times for 10 min with PBS containing 0.1% Triton X-100 under gentle agitation and subsequently incubated for 1 h with secondary antibodies [donkey anti-rabbit Cy3 (Jackson ImmunoResearch, #711-165-152) and donkey anti-goat Cy2 (Jackson ImmunoResearch, #705-225-147)] diluted 1:100 in blocking solution containing 1% donkey serum. Cells were then washed again and mounted in Mowiol overnight until dry.

Image acquisition of single plane images was carried out using an Olympus confocal microscope (Olympus FV3000) with TruSight (deconvolution) technology with the following settings: DAPI 430–470 nm 5% intensity, Alexa Flour488 500–540 nm 2% intensity, Alexa Flour568 570–670 nm 2% intensity. A 100-fold objective (Olympus N5702400) with oil immersion and a numerical aperture of 1.45 was used.

Single-molecule localisation microscopy (SMLM) was accomplished by using dSTORM. dSTORM data were obtained with a Bruker Vutara VXL super-resolution microscope equipped with an ORCA-Fusion BT sCMOS camera, with 405 nm, 488 nm and 555 nm lasers and with the bi-plane detection technique to achieve 3D sub-diffraction resolution. Image processing and movie recording was performed in the SRX software.

All antibodies used for immunofluorescence imaging in this study are listed in Table 1.

**Table 1:** Antibodies used in this study

Antibody	Source	Order number	Species	Dilution
acetylated alpha-Tubulin	Sigma-Aldrich	T6793	mouse monoclonal	1:100
gamma-Tubulin (AAP)	preclinics certified products	D-0006-100UG	goat polyclonal	1:100
gamma-Tubulin (SAS)	preclinics certified products	D-0007-100UG	goat polyclonal	1:100
gamma- Tubulin	Santa Cruz Biotechnology	sc-7396	goat polyclonal	1:100
gamma-Tubulin	Sigma-Aldrich	T6557	mouse monoclonal, GTU-88	1:200
Tmem67	Proteintech	13975-1‐AP	rabbit polyclonal	1:100
Arl13b	Proteintech	17711-1‐AP	rabbit polyclonal	1:100
Rpgrip1l	self-made [33]		rabbit polyclonal	1:200
Anti-goat Dylight488	Jackson ImmunoResearch	705-475-003	donkey	1:100
Anti-mouse Dylight405	Jackson ImmunoResearch	715-475-150	donkey	1:50
Anti-rabbit Cy3	Jackson ImmunoResearch	711-165-152	donkey	1:100

### Antigen preparation

Gamma-tubulin peptide (TUBG1, orb218060, Biorbyt) was conjugated to three different carrier proteins: bovine thyroglobulin (BTG), keyhole limpet hemocyanin (KLH), and cationised bovine serum albumin (cBSA). BTG and KLH were sourced from Sigma-Aldrich, while BSA (Biowest) was cationised using ethylenediamine dihydrochloride and 1-ethyl-3-(3-dimethylaminopropyl)carbodiimide hydrochloride (EDAC-HCl) in 0.1 M MES buffer (pH 4.7). The cationisation reaction was incubated for 2 h at room temperature and then neutralised by washing 20 times with coupling buffer using a 10 kDa MWCO centrifugal filtration column. For peptide conjugation, the peptide and each carrier protein were mixed in equal volumes with EDAC-HCl (final concentration 2 mg/ml) and incubated for 2 h at room temperature. Conjugated samples were dialysed against PBS and stored at − 80 °C until further use. KLH and cBSA conjugates were used for immunization, while the BTG conjugate was used for ELISA-based titre screening.

### Generation of antisera

All animal procedures were conducted in accordance with the German federal animal welfare legislation, specifically Directive 2010/63/EU, the German Animal Welfare Act (TierSchG) and the Animal Welfare Experimental Animal Ordinance (TierSchVersV). All animals were privately owned by Behring Campus Eystrup GmbH which is approved by the competent authorities as an official source for experimental animals. We can confirm that we obtained informed consent from the owners to perform immunisation experiments. One Weiße Deutsche Edelziege (White German Noble Goat) received six subcutaneous injections over a 105-day period, each containing 300 µg of peptide-conjugated carrier protein. KLH and cBSA conjugates were administered in alternating order. Blood samples were collected at three time points: prior to immunization (Day 0, pre-immune serum), two weeks after the third injection (Day 56, first intermediate serum), and two weeks after the fifth injection (Day 98, second intermediate serum) for ELISA-based titre analysis. On Day 119, the goat was euthanized, and the spleen, peripheral blood mononuclear cells (PBMCs) isolated from 150 ml of blood, as well as all available serum were collected for further analysis. Serum collection via final exsanguination by transcutaneous puncture of the jugular vein was performed under appropriate general anesthesia. In ruminants, this is achieved using xylazine and ketamine. According to the officially approved experimental protocol, general anesthesia was administered via intramuscular injection of 0.015 ml xylazine (20 mg/ml) per kg body weight and intravenous injection of 0.1 ml ketamine 10% per kg body weight. The animals were privately owned by another farm which is approved by the competent authorities as an official source for experimental animals.

### Antibody purification and concentration

An antigen affinity column was prepared using the AminoLink™ Plus Immobilization Kit (ThermoFisher, #44894), with 4 mg of gamma tubulin peptide (TUBG1, orb218060, Biorbyt) immobilized using a pH 10 coupling buffer. Goat serum was dialyzed overnight into PBS (pH 7.6, 30 kDa cut-off) and applied to the column in 5 ml aliquots, each incubated for at least 30 min to facilitate binding. Elution was performed with 0.1 M glycine-HCl (pH 3.0) and immediately neutralized with 1 M Tris-HCl (pH 9.0). A total of 16 elution batches were collected, with no evidence of column degradation. Pooled eluates exhibited ELISA titres comparable to the final immunized goat serum collected on Day 119. As an alternative purification method, polyclonal antibodies were isolated by saturated ammonium sulphate (SAS) precipitation using a two-step protocol described earlier [34]. Protein precipitation was initially carried out using 30% ammonium sulphate saturation for 4 h at room temperature. Following centrifugation, the resulting pellet was resuspended in PBS and subjected to a second precipitation step with 45% ammonium sulphate saturation. The mixture was immediately centrifuged, and the pellet was subsequently dissolved in PBS. Finally, the sample was dialyzed against PBS to remove residual ammonium sulphate.

### Enzyme linked immunosorbent assay (ELISA)

ELISA was performed using high-binding 96-well plates (Corning, 100 µl/well), coated overnight at 4 °C with 3 µg/ml of either BTG or BTG-gamma-tubulin in carbonate buffer. After washing with PBS, wells were blocked with I-Block for 2 h at room temperature. Serial dilutions of goat sera from Day 0 (pre-immune), Day 56, and Day 98 were prepared in I-Block (Fisher Scientific, #12684305), starting at 1:50 with a 1:5 dilution series, and incubated for 1 h at room temperature. Bound antibodies were detected using rabbit anti-goat IgG-Fc-HRP (1:10,000 in I-Block), followed by TMB substrate development (Seramun Blau, 15 min, RT), and stopped with 1 M sulfuric acid. The same protocol was applied to assess target-specific binding of the purified antibody.

## Results

**Fig. 1:**
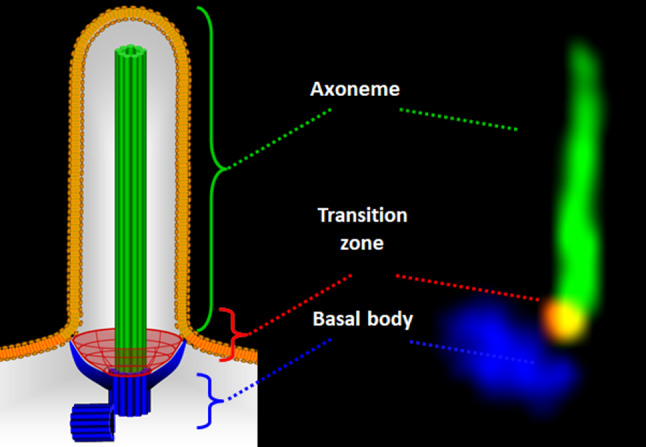
Structure and subcompartments of a primary cilium. (Left) Schematic representation of a primary cilium, highlighting its structural components: the basal body (blue), the transition zone (red), the axoneme (green), and the ciliary membrane (orange). The basal body anchors the cilium to the cell and organizes the microtubules that extend into the axoneme. The transition zone acts as a selective barrier, regulating protein entry and exit, and is critical for ciliary signalling. The axoneme, composed of microtubule doublets, extends from the basal body. The ciliary membrane (orange) is connected to the plasma membrane but contains distinct receptors that mediate cilia-dependent signalling. (Right) Representative multiplex confocal microscopy image of a primary cilium formed by a MEF, with the basal body detected by an anti-gamma-tubulin antibody (Santa Cruz Biotechnology, sc-7396) and stained in blue, the transition zone marked by an anti-RPGRIP1L antibody (self-made [33]), and visualised in red, and the axoneme labelled by an anti-acetylated alpha tubulin antibody (Sigma-Aldrich, T6793) and shown in green. MEFs were fixed in 4% PFA for 1 h at 4 °C

In cilia research and in other disciplines, the visualisation of several proteins at the same time, requires fluorophores with no spectral overlap and ideally primary antibodies from different species. As an example, the three main structural components of the primary cilium, the basal body, the transition zone and the axoneme are often visualized and displayed at the same time (Fig. 1). Very often, suitable antibodies are a limiting factor, because most primary antibodies originate from either rabbit or mouse [35], e.g., commercial anti-gamma-tubulin antibodies from species other than mouse or rabbit are not available. To fill this gap, we set out to produce and evaluate the performance of a novel goat-derived anti-gamma-tubulin antibody designed to enable multiplexing in both basic research and diagnostic applications.

**Fig. 2:**
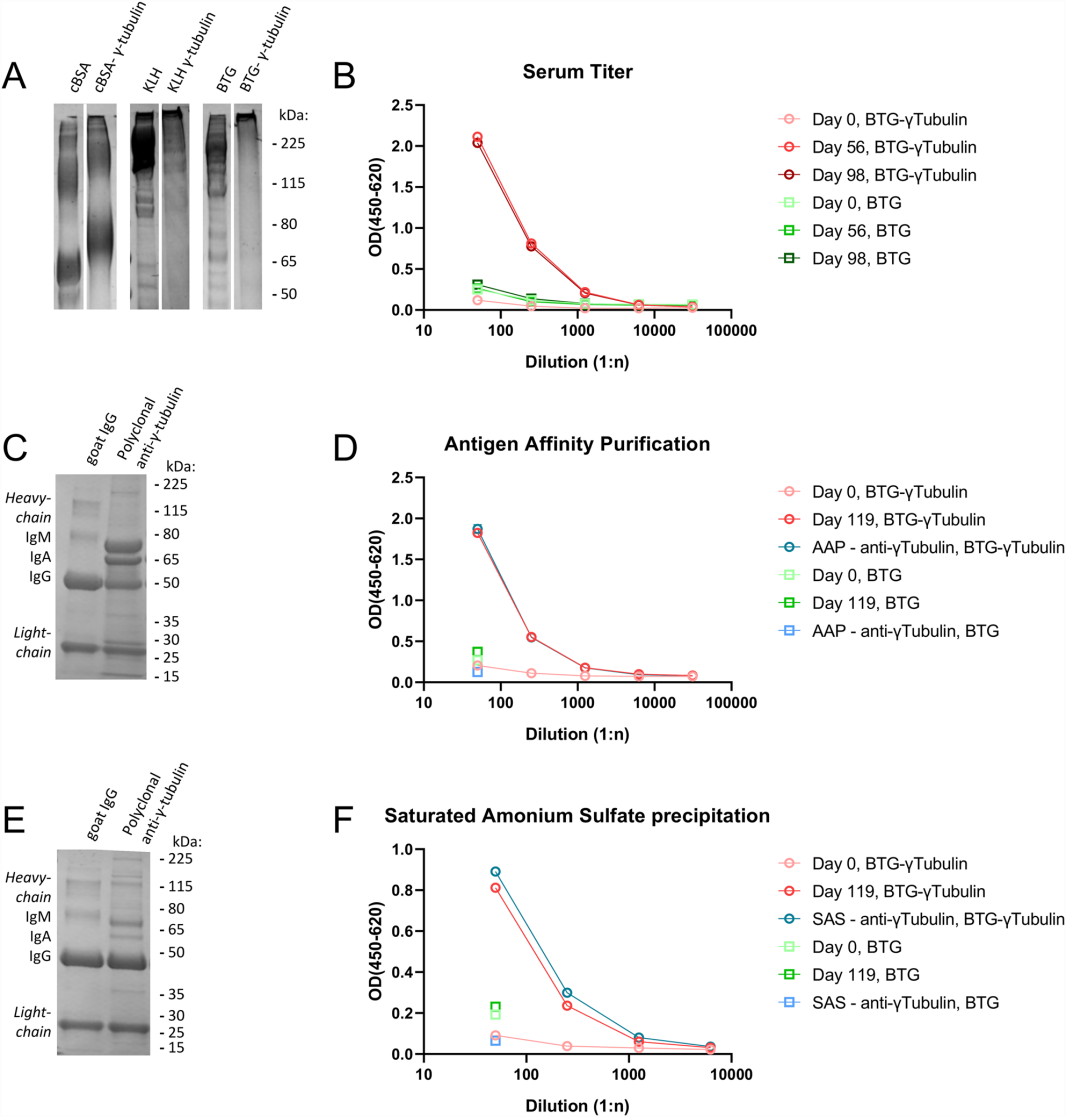
Generation and purification of a gamma-tubulin-specific polyclonal antibody. (**A**) Verification of gamma-tubulin peptide conjugation to carrier proteins. SDS-PAGE analysis of unconjugated carrier proteins (KLH, cBSA, BTG) and their respective gamma-tubulin conjugates. (**B**) ELISA analysis of serum titre during immunization. A goat was immunized alternately with KLH- and cBSA-conjugated gamma-tubulin. Intermediate serum collected on Day 56 (after three immunizations) and Day 98 (after five immunizations) shows strong reactivity to BTG-conjugated gamma-tubulin but not to unconjugated BTG. Pre-immune serum (Day 0) shows no detectable binding. (**C**) SDS-PAGE of affinity-purified polyclonal antibody. Final serum collected on Day 119 (after six immunizations) was dialyzed and subjected to antigen-specific affinity purification. The gel shows bands corresponding to antibody light chains and putative heavy chains, consistent with the molecular weight of IgG, IgA, and IgM isotypes. (**D**) ELISA confirmation of reactivity following antigen-specific affinity purification. The affinity-purified antibody retains strong and specific binding to BTG-conjugated gamma-tubulin, with signal comparable to unpurified Day 119 serum. (**E**,** F**) ELISA of antibody preparation after saturated ammonium sulphate (SAS) precipitation. The SAS-precipitated antibody preparation shows strong reactivity to BTG-conjugated gamma-tubulin, similar to unpurified Day 119 serum, confirming effective enrichment of antigen-specific antibodies. E is SDS-PAGE, F is ELISA

To generate the antibody, a gamma-tubulin peptide was conjugated to three different carrier proteins: keyhole limpet hemocyanin (KLH), cationised bovine serum albumin (cBSA), and bovine thyroglobulin (BTG). Successful conjugation was verified by SDS-PAGE, showing a distinct shift in protein size compared to the unconjugated carrier proteins (Fig. 2A). One goat was immunized alternately with KLH- and cBSA-conjugated gamma-tubulin. Intermediate sera collected on Day 56 (after three immunizations) and Day 98 (after five immunizations) specifically recognized BTG-conjugated gamma-tubulin in ELISA, while showing no significant reactivity to unconjugated BTG (Fig. 2B). Similarly, pre-immune serum did not show binding to the BTG-gamma-tubulin conjugate.

Final serum collected on Day 119 (after six immunizations) was dialyzed against PBS and subjected to antigen-specific affinity purification. SDS-PAGE analysis of the purified polyclonal antibody revealed characteristic light chain bands and multiple heavy chain bands corresponding in size to immunoglobulin isotypes IgG, IgA, and IgM (Fig. 2C). ELISA confirmed that the purified antibody maintained strong and specific reactivity to BTG-conjugated gamma-tubulin, comparable to the signal observed with the unpurified final serum from Day 119 (Fig. 2D).

In addition, a polyclonal antibody preparation obtained via saturated ammonium sulphate (SAS) precipitation also showed strong and specific binding to BTG-conjugated gamma-tubulin, with reactivity comparable to that of the unpurified Day 119 serum (Fig. 2E).

**Fig. 3:**
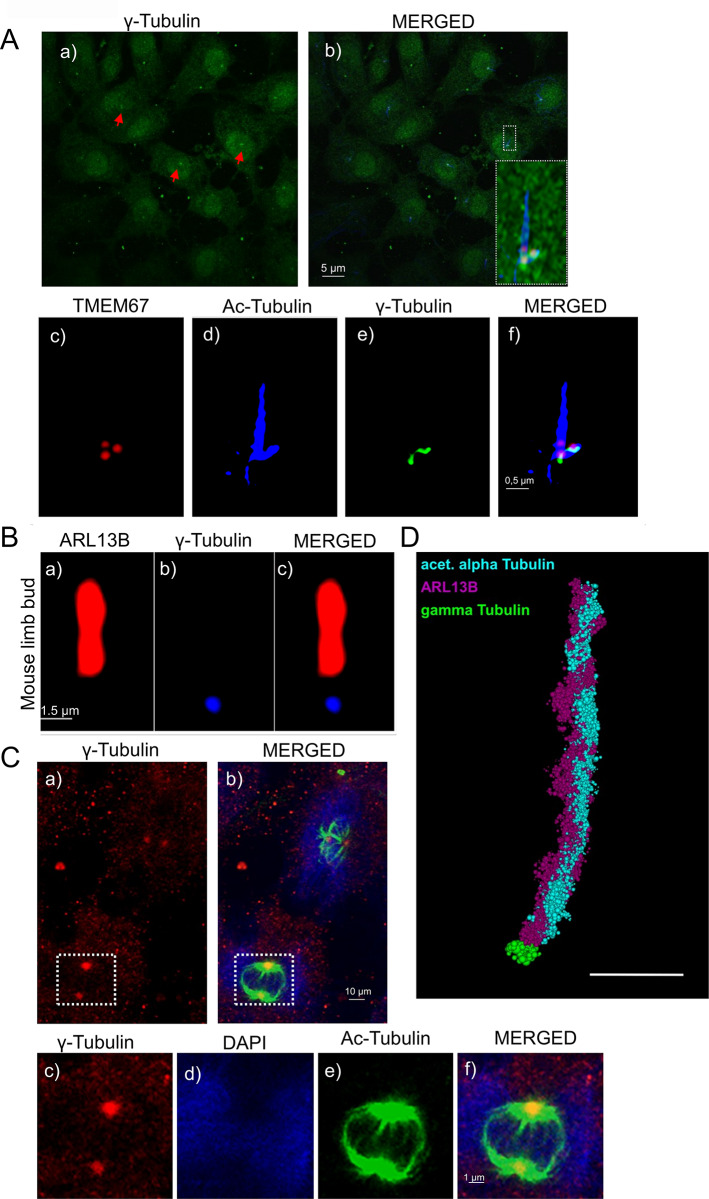
Performance of SAS precipitated gamma-Tubulin Antibody. (**A**) Immunofluorescence staining showing SAS precipitated gamma-Tubulin (# D-0007-100UG) performance in ciliated NIH-3T3 cells. (**a**) Red arrows highlight basal body/centriole positions in different cells. (**b**) Merged images including gamma-Tubulin (green), anti-acetylated tubulin (blue), and anti TMEM67 (red). The white square outlines a representative ciliary structure used for higher magnification. Image in the right corner represents original non enhanced signal. (**c-f**) Magnified and digitally enhanced area from b) showing co-localization of TMEM67 (red), acetylated α-Tubulin (blue), and gamma-Tubulin (green). The merged panel demonstrates the spatial relationship of the different ciliary subcompartments. (**B**) Magnified and digitally enhanced immunofluorescence staining of a primary cilium in the mouse limb bud shows the ciliary membrane protein ARL13B (red, **a**) and gamma-tubulin (blue, **b**) marking the basal body. The merge panel illustrates the spatial relationship between both subcompartments (**c**). Scale bar represents 1.5 μm. (**C**) Immunofluorescence staining of mitotic NIH-3T3 cells. (**a**) γ-Tubulin staining (red) highlights centrosomes/spindle poles. (**b**) Merged image including γ-Tubulin (red), acetylated α-Tubulin (green), and DAPI (blue). The inset shows a magnified region illustrating γ-Tubulin localization at centrosomes/spindle poles during mitosis (**c**-**f**). Scale bars as indicated (**D**) Immunofluorescence was performed on serum-deprived NIH-3T3 cells. Super-resolution image was obtained by using dSTORM. The ciliary axoneme is shown in turquoise, labelled by acetylated alpha-Tubulin; segments of the ciliary membrane appear in magenta, marked by ARL13B; and the basal body is depicted in green through staining with SAS precipitated gamma-Tubulin (# D-0007-100UG). The scale bar (in white) represents a length of 1 μm. The cilium is shown in point cloud mode

**Fig. 4:**
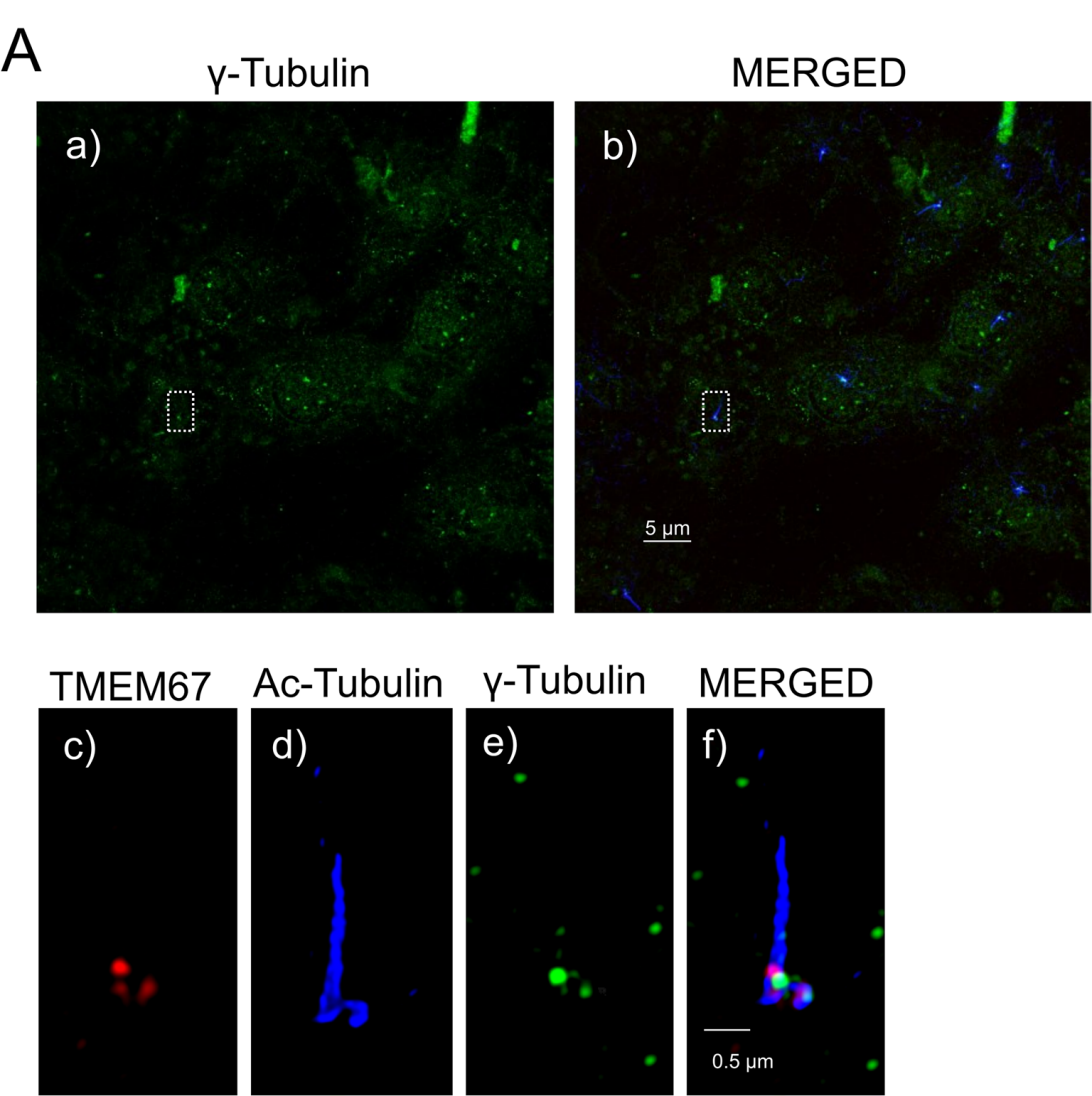
Performance of AAP purified gamma-Tubulin Antibody. (**A**) Immunofluorescence staining showing AAP purified gamma-Tubulin (#D-0006-100UG) performance in ciliated NIH-3T3 cells. Merged images including gamma-Tubulin (green), anti-acetylated tubulin (blue), and anti TMEM67 (red). The white square outlines a representative ciliary structure used for higher magnification in c-f. Scale bar represents 1 μm

To evaluate the specificity and performance of the newly generated gamma-tubulin polyclonal antibody (SAS precipitated) in cellular imaging, immunofluorescence staining was first performed on serum-starved and methanol fixed NIH-3T3 cells. Staining with gamma-tubulin antibody revealed distinct punctate green signals at the base of the cilium, consistent with basal body localization. Red arrows highlight several basal body positions in each cell of the image section, indicating consistent detection across multiple cells (Fig. 3A, a). Co-immunostaining with antibodies against acetylated α-tubulin (blue), a marker of the axoneme, and TMEM67 (red), a known transition zone (TZ) protein, further validated the basal body specificity of the gamma-tubulin antibody. The merged image shows a clear spatial separation of these markers at the corresponding sites (Fig. 3A, b). Higher magnification and digitally enhanced images of the selected ciliary structures demonstrates precise localization of all three markers at the expected sites (Fig. 3A, c-f). The same images without digital enhancement are shown in Figure S2. Similar pattern was observed with gamma-tubulin antibody that was purified by affinity purification (AAP, D-0006-100UG), however with an overall weaker signal (Fig. 4). The antibody allows robust labelling of centrioles and spindle pole structures in mitotic cells (Fig. 3C). Further, it is applicable to visualize primary cilia using super-resolution microscopy (dSTORM) (Fig. 3D**)**. To critically test the impact of fixation on immunostaining performance of the new antibody, we additionally used paraformaldehyde (PFA) fixation. Notably, NIH-3T3 cells fixed with 4% PFA at 4 °C for 1 h failed to exhibit any detectable staining of basal bodies (not shown). This indicates that PFA fixation strongly impacts antigen accessibility and masks relevant epitopes by crosslinking. Interestingly, cryosections of embryonic mouse limb buds that were initially fixed with PFA did not show basal body staining at first, but, when subsequently treated with methanol fixation, distinct basal body staining was visible (Fig. 3B). This suggests that methanol fixation on samples previously fixed with PFA can partially restore antigen accessibility and open the possibilities for optimizing fixation methods for individual purposes. To further critically test the performance of the novel antibody we included strategies for antibody validation proposed by Uhlen and coworkers and compared the staining pattern of the novel goat antibody by co-staining with another well tested gamma-tubulin antibody (Sigma-Aldrich, T6557, mouse) [36]. Both signals show robust co-localisation demonstrating specificity (Figure S3A). Similarly, the preincubation with a gamma tubulin blocking peptide reduced the signal compared to the control (Figure S3B).

## Discussion

The generation of a novel goat-derived polyclonal antibody targeting gamma-tubulin represents a significant advancement for multiplex immunofluorescence imaging of the basal body and related microtubule-organizing structures like centrioles. The demand for such an antibody became particularly obvious, after Santa Cruz Biotechnology discontinued the production and sales of their widely used goat polyclonal gamma-tubulin (RRID: AB_2211262; Santa Cruz Biotechnology Cat# sc-7396) in 2016. This antibody had been used in several high impact studies for the visualization of basal bodies or centrioles in immunofluorescence studies with multiplexing purposes [37–40], showcasing its importance. By producing and validating a new goat derived gamma-tubulin antibody, we address this gap and provide an alternative tool especially for multi-channel imaging of basal bodies in cilia research.

Our antibody demonstrated strong specificity and consistent signal at basal bodies in methanol-fixed cells, aligning with the expected localization of gamma-tubulin. Co-staining with antibodies against acetylated-α-tubulin, TMEM67 and ARL13b confirmed its compatibility with various rabbit- and mouse-derived antibodies. Importantly, the goat-derived antibody also performed reliably well in embryonic tissue sections, enabling its utility in developmental studies and in pathology. A critical factor influencing the success of gamma-tubulin detection was the choice of fixation method. Methanol fixation consistently enabled robust and specific staining of basal bodies and centrioles, while paraformaldehyde (PFA) fixation abolished the signal, presumably due to cross-linking of epitopes and impaired antigen accessibility. We observed that sections of mouse limb buds initially fixed with PFA became reactive upon subsequent methanol treatment, restoring basal body staining (Fig. 3B). These findings underscore the sensitivity of gamma-tubulin epitope recognition to fixation conditions and suggest that methanol can partially reverse PFA-induced epitope masking. To further improve antibody performance, we propose that optimization of fixation protocols, e.g. by reducing PFA concentration or fixation duration, can enhance antigen accessibility and signal intensity. In addition, established epitope unmasking techniques such as heat-induced epitope retrieval (HIER) and proteolytic-induced epitope retrieval (PIER) may be promising strategies to test in combination with PFA fixation [35].

We also compared two purification strategies of the serum and used antigen-specific affinity purification (AAP) and saturated ammonium sulfate (SAS) precipitation to concentrate antibodies. AAP provided highly specific antibody preparations, with low background and strong signal retention (Fig. 4). Similarly, SAS precipitated antibodies showed a slightly increased background signal and a distinct and specific signal at the basal body. Both protocols produced antibody preparations with comparable ELISA reactivity and specificity (Fig. 2).

Novel techniques such as expansion microscopy (ExM) offer enhanced resolution using standard confocal laser scanning microscopy [41]. These approaches have significantly advanced our ability to visualize sub-compartmental ciliary structures [42, 43]. ExM protocols are typically optimized for paraformaldehyde PFA-fixed specimens; however, adaptations for methanol-fixed samples have also been reported e.g., in *Chlamydomonas reinhardtii* [44]. This flexibility in fixation compatibility suggests that our goat-derived gamma-tubulin antibody, may be suitable for integration into ExM protocols as well.

Gamma-tubulin is an evolutionary conserved component of the microtubule-organizing center (MTOC) and plays a central role in mitotic spindle assembly, centrosome duplication, and microtubule nucleation [31, 45]. Aberrations in centrosomal proteins, including gamma-tubulin, are implicated in various cancers [46, 47] as well as in neurodevelopmental disorders like microcephaly and others [48]. Consequently, the novel antibody can also serve as a valuable tool for research in oncology and neurodevelopmental disorders involving centrosomal dysfunction.

## Conclusions

Taken together, in this study, we address a key limitation in multiplex immunofluorescence imaging of primary cilia, particularly basal bodies and related structures, by developing a gamma tubulin antibody raised in goat. This antibody demonstrates strong specificity and performance in labelling basal bodies and centrioles. Its goat origin enables flexible and comprehensive multiplex staining strategies alongside established ciliary markers.

## Supplementary Information

Below is the link to the electronic supplementary material.

Supplementary Material 1: Figure S1: Uncropped images corresponding to Figure 2. (A) Full, uncropped gel image corresponding to the cropped panels shown in Figure 2A. Colored dashed boxes indicate the regions that were selected and presented in the main figure. Molecular weight markers (kDa) are indicated. (C) Uncropped gel images corresponding to Figure 2C. The red dashed box highlights the portion of the blot used in the main figure. Molecular weight markers (kDa) are indicated. (E) Full gel image corresponding to Figure 2E. The red dashed box indicates the region shown in the main figure Molecular weight markers (kDa) are shown

Supplementary Material 2: Figure S2: Performance of SAS precipitated gamma-Tubulin Antibody. (A) Magnified and non-digitally enhanced images from Figure 3A, panel c-f

Supplementary Material 3: Figure S3: Specificity of SAS precipitated gamma-Tubulin Antibody. (A) Immunofluorescence images showing staining of ARL13B and γ-tubulin in cells. (a) ARL13B labeling (red) highlights the ciliary membrane. (b) γ-Tubulin detected with mouse monoclonal antibody (Sigma Aldrich, T6557, blue) marks the centrosome/basal body. (c) γ-Tubulin with goat polyclonal antibody (preclinics D-0007, green) used to validate the specificity of the newly generated antibody. (d) Merged image showing the spatial relationship between ARL13B and γ-tubulin signals. The γ-tubulin signal detected by the new antibody (green) co-localizes with the established mouse γ-tubulin marker at the centrosome, supporting the specificity of the new antibody. (B) Peptide blocking assay demonstrating the specificity of the SAS precipitated γ-Tubulin antibody. Immunofluorescence images show ARL13B (red) and γ-Tubulin detected with the goat polyclonal antibody (preclinics D-0007, green) in the absence (− blocking peptide) and presence (+ blocking peptide) of the immunizing peptide. In the absence of blocking peptide, γ-Tubulin signal is detected at the basal body and co-localizes with the base of the ARL13B-positive cilium. Upon addition of the blocking peptide, the γ-Tubulin signal is strongly reduced or abolished, while ARL13B staining remains unaffected. Scale bar: 1 µm

## Data Availability

All data of this study are available from the corresponding author upon request. The goat-derived gamma tubulin antibodies (gamma-Tubulin (AAP); D-0006-100UG and gamma-Tubulin (SAS); D-0007-100UG) developed in this study can be purchased directly from preclinics certified products GmbH.
