# Supplementary figures and images for: A goat-derived gamma-tubulin antibody for triple-channel imaging of primary cilia
Source: BMC Mol Cell Biol. 2026 May 28;27:31. doi: 10.1186/s12860-026-00595-7 (PMC13217945; doi:10.1186/s12860-026-00595-7)

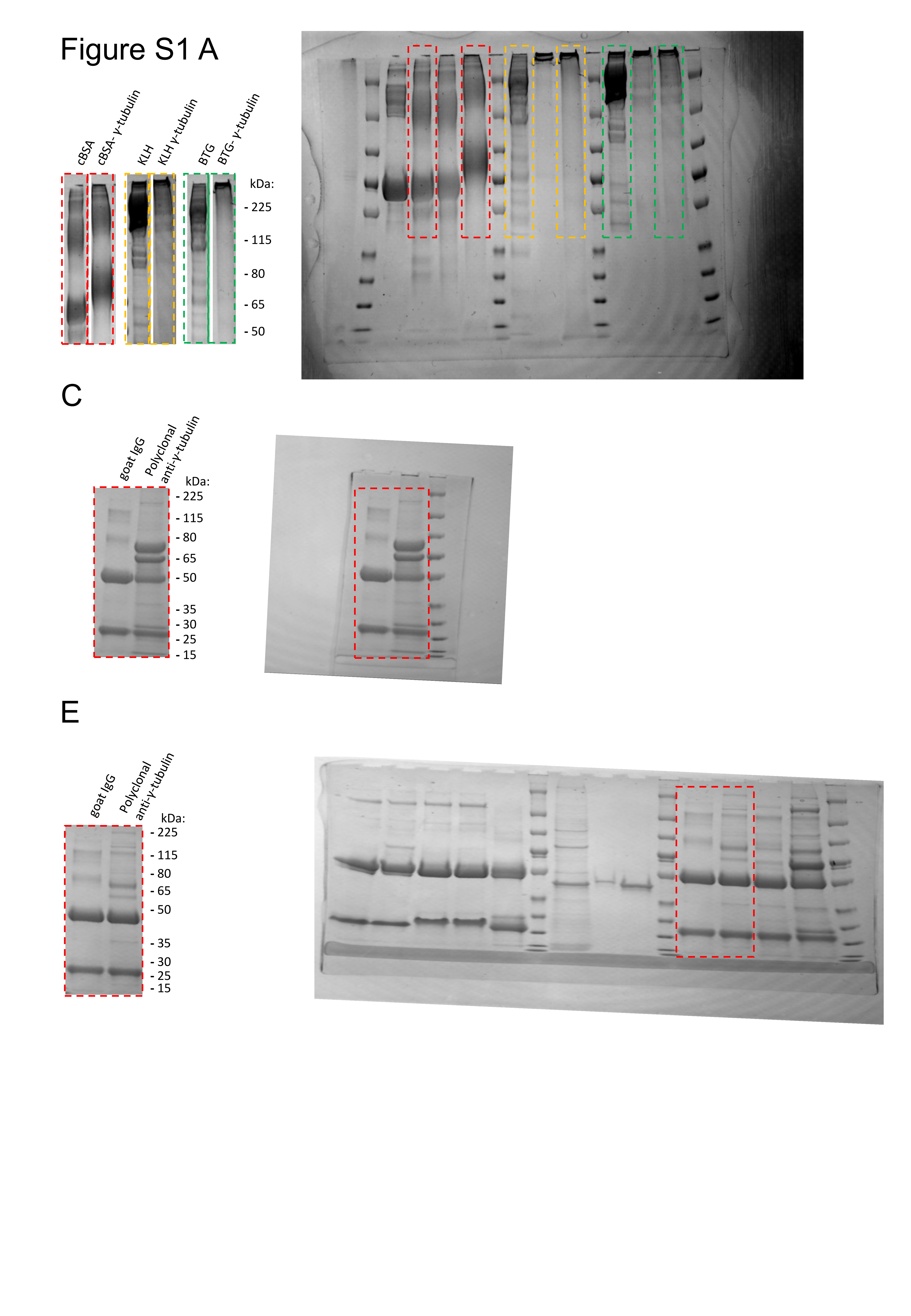

Supplement: Supplementary file 1 — Supplementary Material 1: Figure S1: Uncropped images corresponding to Figure 2. (A) Full, uncropped gel image corresponding to the cropped panels shown in Figure 2A. Colored dashed boxes indicate the regions that were selected and presented in the main figure. Molecular weight markers (kDa) are indicated. (C) Uncropped gel images corresponding to Figure 2C. The red dashed box highlights the portion of the blot used in the main figure. Molecular weight markers (kDa) are indicated. (E) Full gel image corresponding to Figure 2E. The red dashed box indicates the region shown in the main figure Molecular weight markers (kDa) are shown [file 12860_2026_595_MOESM1_ESM.png]

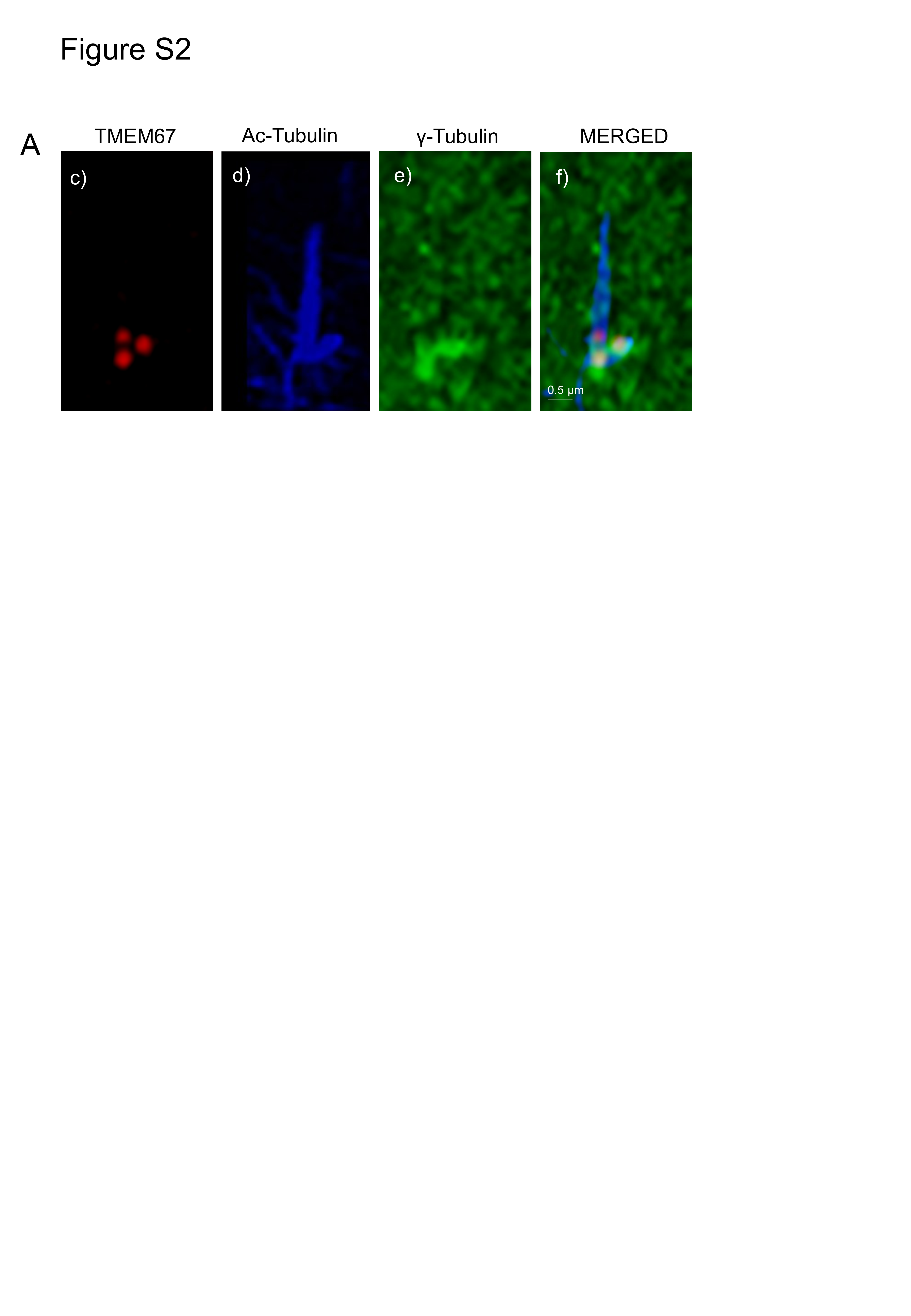

Supplement: Supplementary file 2 — Supplementary Material 2: Figure S2: Performance of SAS precipitated gamma-Tubulin Antibody. (A) Magnified and non-digitally enhanced images from Figure 3A, panel c-f [file 12860_2026_595_MOESM2_ESM.png]

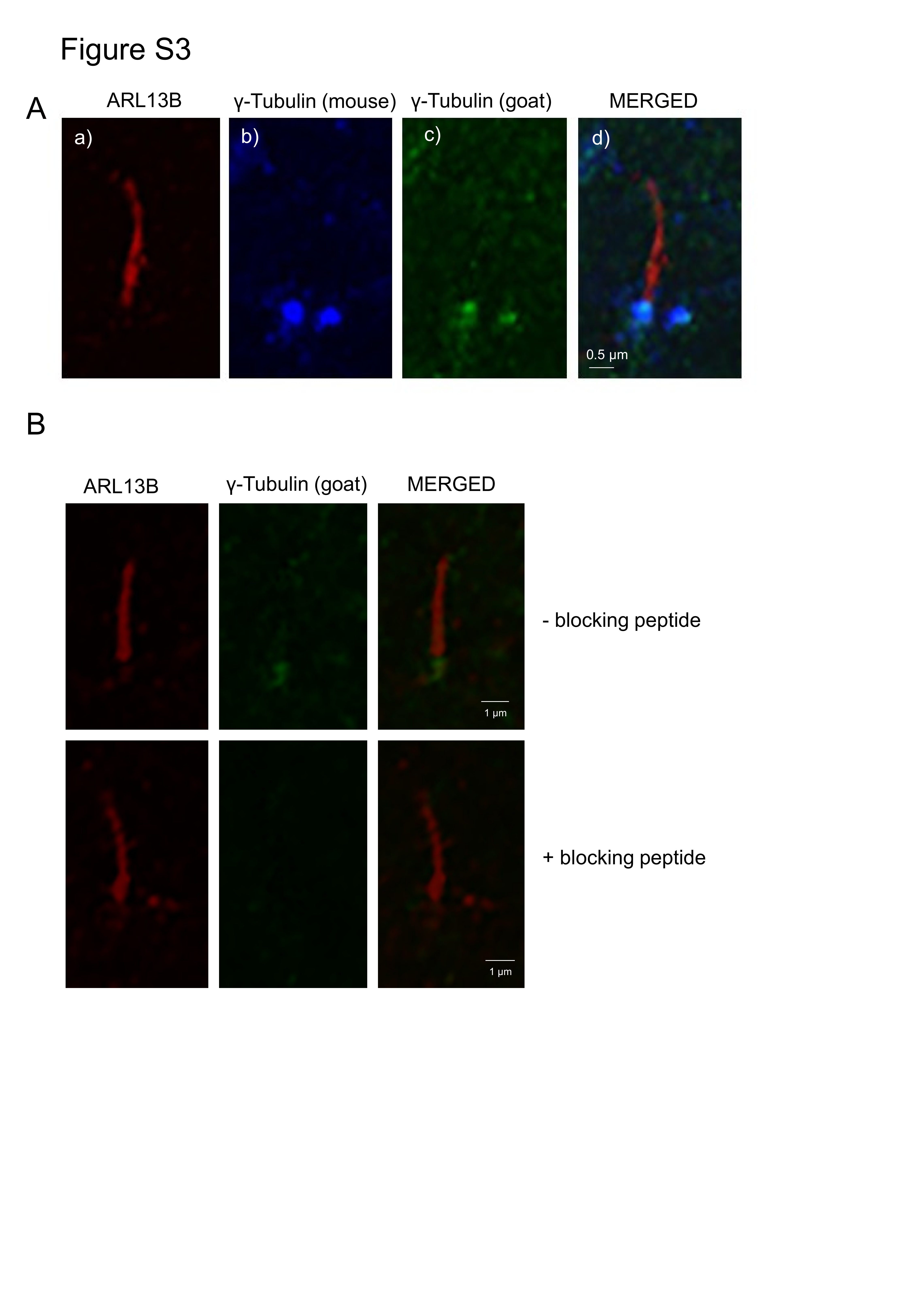

Supplement: Supplementary file 3 — Supplementary Material 3: Figure S3: Specificity of SAS precipitated gamma-Tubulin Antibody. (A) Immunofluorescence images showing staining of ARL13B and γ-tubulin in cells. (a) ARL13B labeling (red) highlights the ciliary membrane. (b) γ-Tubulin detected with mouse monoclonal antibody (Sigma Aldrich, T6557, blue) marks the centrosome/basal body. (c) γ-Tubulin with goat polyclonal antibody (preclinics D-0007, green) used to validate the specificity of the newly generated antibody. (d) Merged image showing the spatial relationship between ARL13B and γ-tubulin signals. The γ-tubulin signal detected by the new antibody (green) co-localizes with the established mouse γ-tubulin marker at the centrosome, supporting the specificity of the new antibody. (B) Peptide blocking assay demonstrating the specificity of the SAS precipitated γ-Tubulin antibody. Immunofluorescence images show ARL13B (red) and γ-Tubulin detected with the goat polyclonal antibody (preclinics D-0007, green) in the absence (− blocking peptide) and presence (+ blocking peptide) of the immunizing peptide. In the absence of blocking peptide, γ-Tubulin signal is detected at the basal body and co-localizes with the base of the ARL13B-positive cilium. Upon addition of the blocking peptide, the γ-Tubulin signal is strongly reduced or abolished, while ARL13B staining remains unaffected. Scale bar: 1 µm [file 12860_2026_595_MOESM3_ESM.png]
